# Remnant hollowed out dead coral skeleton branches defer coral community recovery

**DOI:** 10.1371/journal.pone.0339527

**Published:** 2026-03-11

**Authors:** Kathryn C. Scafidi, Kyle W. Fouke, Mayandi Sivaguru, Bruce W. Fouke, Peter J. Edmunds

**Affiliations:** 1 Department of Biology, California State University, Northridge, California, United States of America; 2 Department of Geoscience, University of Wisconsin Madison, Madison, Wisconsin, United States of America; 3 Department of Earth Science & Environmental Change, University of Illinois Urbana-Champaign, Urbana, Illinois, United States of America; 4 Cytometry and Microscopy to Omics Facility, Roy J. Carver Biotechnology Center, University of Illinois Urbana-Champaign, Urbana, Illinois, United States of America; 5 Cancer Center at Illinois, University of Illinois Urbana-Champaign, Urbana, Illinois, United States of America; 6 Department of Evolution, Ecology and Behavior, School of Integrative Biology, University of Illinois Urbana-Champaign, Urbana, Illinois, United States of America; 7 Roy J. Carver Biotechnology Center, University of Illinois Urbana-Champaign, Urbana, Illinois, United States of America; University of the Ryukyus, JAPAN

## Abstract

Through repeated impacts of ecosystem disturbances, most coral reefs have transitioned to a degraded state with low living coral cover. Until recently, the reefs of Moorea, French Polynesia, have provided an exception to this trend as their coral communities have recovered from sequential disturbances over the last 50 years. Early in 2019, the north shore fore reef at 10-m depth had ~ 75% live coral cover, but was decimated by bleaching to leave 17% coral cover by August 2020 and many kilometers of reef dominated by dead-in-place colonies of *Pocillopora* spp. By 2025, coral recovery had not begun because of the chronology of decay affecting dead *Pocillopora* skeletons. We combine ecological analyses of dead *Pocillopora* colonies with high-resolution microscopy of aragonite skeletal structure to better understand the fate of these dead branching corals. Bleaching in 2019 created a reef dominated by dead *Pocillopora* colonies that initially were resilient to breakage and removal. Dead corals were colonized by macroalgae, occupied by a unique assemblage of invertebrates, and deterred coral recruitment as evidenced by coral settlers on settlement tiles but not on dead skeletons. In 2022–2023, three years after *Pocillopora* colonies were killed, their remnant hollowed-out branches were first detected. High-resolution microscopy indicates hollowing occurred through bioerosion of dead branches that were scaffolded by encrusting taxa and entrapped sediments. Over 2011–2019, coral communities in Moorea rapidly recovered from devastation, but recovery from the 2019 bleaching has been deferred by the retention of dead corals on reef surfaces, creating the opportunity for erosion to further hollow out and weaken dead coral branches. This study shows how the synergy of well-studied disturbances can create ecological surprises that at best defer community recovery, but at worst portend a different outcome of major disturbance events, preventing recovery of the original coral community.

## Introduction

Structurally complex ecosystems are often characterized by high species diversity [1,2] because they are relatively stable environments that provide abundant niches in which organisms can successfully adapt, acclimatize, evolve and thrive [3]. In marine ecosystems, temperate giant kelp forests and tropical coral reefs provide a structural framework that is foundational to establishing ecosystem species diversity [4–6]. The kelp *Macrocystis pyrifera* grows to > 30 m in height in temperate seas and supports a variety of species that utilize the layered canopy and understory habitats [7–11]. In similar ways, corals contribute structural elements to shallow marine communities, and while these elements are smaller than those provided by giant kelps, they support high species diversity [12–14]. Branching corals in particular host a high diversity of organisms among and beneath their branches [13,15–17]. While the structural elements of living biota can play pivotal roles in defining ecosystem features [18], these emergent properties are subject to rapid loss through disturbances such as diseases [19,20], storms [21–24], and predator outbreaks [25].

Regular ecosystem disturbances of moderate magnitude that are caused by storms, disease and other factors can promote coral reef species diversity by preventing spatial dominance by a single taxon and creating vacant space for recruitment [26]. In communities dominated by sessile taxa, the detachment of dead organisms from the substratum and the removal of their debris often is necessary to create vacant spaces that are essential for recruitment and recovery [27]. Coral reefs are a dynamic ecosystem in which structural complexity [28] is dependent upon scleractinian corals that are experiencing widespread mortality through climate change and other anthropogenic disturbances [29]. Branching corals are common on reefs in the Indo-Pacific [30], where they create structural complexity that is periodically affected by disturbances due to population explosions of the crown-of-thorns sea star (COTs, *Acanthaster* spp.) [31], storms [32], and coral bleaching [33]. Storms can remove corals regardless of whether they are alive or dead when the storm arrives [34], but COTs and bleaching leave dead corals in growth position until they are removed by erosion or storms [35,36]. When dead corals are detached from the reef, the rubble they produce can create persistent legacy effects (sensu [37]) that can propagate damage across the reef through burial and abrasion [38]. Yet coral rubble can serve important ecological functions including facilitating coral recruitment when the rubble has been consolidated [39].

The recovery dynamics following disturbances plays an important role in determining the kind of coral community that develops [40,41]. Fast-growing, “weedy” corals [42] often are the first settlers to vacant space, and this functional group is well represented throughout the Indo-Pacific by branching *Pocillopora* spp. (hereafter *Pocillopora*) [43]. Members of this genus are early colonizers of vacant space [44], they grow rapidly (roughly 1.5–4.5 cm y^-1^) [45,46] and can spatially dominate benthic surfaces [47]. For the last several decades, this has been the case on the fore reef of Moorea, French Polynesia, where *Pocillopora* have dominated coral communities following a COTs outbreak and cyclone in 2010 [44,48]. Live *Pocillopora* reached 58% cover at 10-m depth on the north shore fore reef by March 2019 [49] but bleaching in April 2019 reduced cover to 12% by 2020 and left dead *Pocillopora* colonies attached to the reef. The recent history of these reefs creates the opportunity to test the role of non-living structural elements in mediating the recovery of a coral reef on which previous disturbances support an expectation of rapid coral community recovery [44,48].

**Fig 1:**
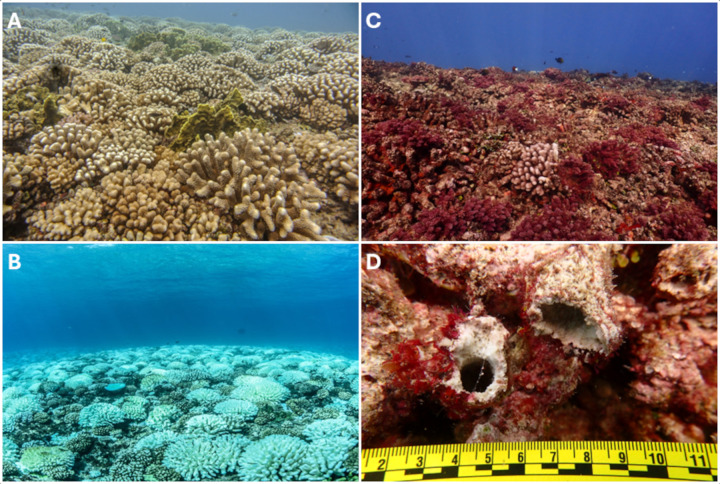
Images of the study reef and hollow corals in 2024. The fore reef of Moorea at 10-m depth showing the high coverage of live *Pocillopora* coral before **(A)**, during **(B)**, and after **(C)** the 2019 bleaching. Only a few live *Pocillopora* remained on the reef by 2024 (e.g., center foreground of **(C)**), and the reef was spatially dominated by dead corals encrusted by the macroalga *Lobophora variegata* and tufts of the red macroalga *Asparagopsis taxiformis*. **(D)** Naturally fractured branches of *Pocillopora* showing the internal hollowing and the profusions of encrusting taxa hypothesized to provide a scaffolding effect allowing the internal dissolution to proceed.

The reefs of Moorea have been scientifically studied since the 1970s [50] thereby codifying the expected chronology of events affecting coral communities following major disturbances (e.g., [44,48,51]). Unlike many contemporary coral reefs, the fore reef of Moorea has shown high resilience to disturbances [44,48,51], with episodes of large-scale coral mortality followed by rapid colonization and regrowth of corals within 3–5 years of near total loss [47]. We took a traditional ecological approach to assess the effects of dead-in-place skeletons on the post-disturbed coral reef community and supplemented the novel discovery of hollowing branches using analysis of aragonite minerology. First, the loss of *Pocillopora* colonies was quantified by tagging and monitoring dead and live colonies over one year (2022–2023). The appearance of vacant space on the benthos surrounding tagged colonies was quantified to evaluate the processes promoting coral recruitment. Second, we contrasted the assemblages of invertebrates within dead and live *Pocillopora* colonies and indirectly tested their effects by comparing the benthic community structure < 70 cm from live and dead colonies. We hypothesized that population increases of micro-herbivores like amphipods [52] among the branches of dead corals would affect the algal community structure on the adjacent benthos [53]. Third, following the discovery of hollow branches in dead *Pocillopora* colonies (Fig 1), we explored the role of branch hollowing in mediating the breakage of dead skeletons. Once fractured, hollow branches were visible in a planar view and could be quantified using photoquadrats (0.5 × 0.5 m). Prior to fracturing, hollow branches were quantified *in situ* using a “crush assay”, and voucher specimens were used to describe their mode of formation through microscopic analysis of their mineralogy. Finally, we interpret the ecological significance of dead-in-place *Pocillopora* colonies by contrasting the trajectories of coral community recovery over two 5-year periods, one initiated by cyclone Oli in 2010 [44,48,51] and one initiated by bleaching in 2019.

## Materials and methods

### Study sites

Fore reef ecosystems on the north shore of Moorea, French Polynesia (two sites, LTER 1 17° 28.501’ S, 149° 50.224’ W and LTER 2 17° 28.281’ S, 149° 48.489’ W) (S1 Fig [54,55]) were studied in May 2022 through April 2024, while working from the University of California Richard B. Gump Research Station. Long-term monitoring of these well-studied reefs [56–60] permits quantification of the ecological effects of two primary types of globally impactful marine ecosystem disturbances. The first was a cyclone in February 2010 that removed dead coral skeletons killed by COTs predation [61], which produced large areas of rocky submarine hardgrounds. The other was a bleaching event in 2019 [62,63] that left abundant dead in-place coral colonies (mostly *Pocillopora*) that still had not been recolonized by living corals in April 2024. Photoquadrats (0.5 × 0.5 m) have been documented as part of the long-term analyses of these reefs since the start of the LTER in 2005 where a single, permanently marked transect (50 m long) at 10-m depth at LTER 1 and LTER 2 (S1 Fig) was established. Photoquadrats were randomly positioned along the transect when the study began and were thereafter photographed at the same point on the transect in April each year (n = 40 photoquadrats year^-1^ at each site). The photoquadrats were recorded digitally using a camera mounted on a framer held perpendicular to the substratum (described in [49,51]) and the camera was attached to two strobes (Nikonos SB105). The photoquadrats were analyzed using CoralNet software with 200 randomly located dots annotated manually for the benthic space holders on which they were located. This study was carried out under the field permit DIREN-2907, Protocole d’Accueil 2022–2023 & 2023–2024, granted by the French Polynesian Government (Délégation à la Recherche).

### Ecological approaches

In May 2022, to evaluate the fate of *Pocillopora* colonies, dead (n = 5) and live (n = 30) colonies were randomly selected and tagged parallel to the established long-term 50-m transect at LTER 1 at 10-m depth. The fate of the tagged colonies was assessed through four repeated surveys over a year. When the tagged colonies were found, they were lightly touched to determine if they were detached from the underlying reef rock. Colonies were scored as found or as missing when they could not be detected after scouring the benthos (i.e., they were presumed to have become detached from the reef between samplings).

Due to the limited remaining live *Pocillopora* colonies on the reef, distance between tagged colonies along the transect varied, however, care was taken to tag neighboring colonies over 1 m away from each other so there was no overlap of benthic photographs. Photos covering 1 m^2^ areas centered on the tagged colonies (four 0.5 × 0.5 m quadrats per colony to ensure high resolution of benthos) were captured for benthic characterization described below using an Olympus TG6 camera (12 megapixels) held in planar view above the quadrats. Photoquadrats were recorded using available light and were white balanced against a white slate at depth using the manufacturer’s settings on the camera. Surveys were repeated in August 2022, January 2023, and April 2023 to determine the percentages of detached colonies, which were compared between dead and live colonies and across all sampling periods using a Fisher’s exact test (2 × 4 contingency table of Condition × Time) using the R base function fisher.test() from the *stats* package [64]. Additionally, the fate of dead *Pocillopora* was indirectly evaluated by measuring areas of vacant patches on the reef, with the assumption that removal of adjacent dead *Pocillopora* would increase the size of existing patches or create new patches as dead colonies of *Pocillopora* dislodge. Analysis of vacant patches were completed using the photoquadrats of the 35 tagged colonies in May 2022, August 2022, January 2023, and April 2023, using ImageJ software [65]. Vacant space within 1 m^2^ area around dead and live corals was expressed as percentage of the open substratum (i.e., not occupied by living organisms), and this was compared between colony conditions (live versus dead) using a beta-family generalized linear mixed-effects model (GLMM) with a logit link (glmmTMB, R package: *glmmTMB* [66]). Fixed effects were condition and time (May 2022, August 2022, January 2023, and April 2023) and their interaction (Condition × Time), which tested whether changes in vacant space across time differed between live and dead colonies. To account for the repeated measures on individual colonies, Coral ID was included as a random effect. A model including random slopes was evaluated but failed to converge due to overparameterization, thus the model used included random intercepts only. Model assumptions were assessed using simulation-based residual diagnostics with the *DHARMa* package in R [67]. There were no significant deviations from the expected uniform distribution (Kolmogorov–Smirnov p = 0.70) or any evidence of overdispersion (p = 0.34), therefore the model assumptions were met.

To test the hypothesis that invertebrate assemblages differ between dead and live *Pocillopora*, colonies were sampled for invertebrates within their coralla. Dead (n = 4) and live (n = 4) colonies were collected from 10-m depth across two years. Sampled colonies were < 20-cm diameter and were collected using a hammer and chisel from outside the survey areas used to evaluate colony fate. Freshly detached colonies were placed in sealable plastic bags underwater to retain associated invertebrates and were returned to the lab where they were processed on the day of collection.

The seawater in each bag was filtered through 110 µm mesh to retain loose invertebrates, and the colonies were rinsed with freshwater to dislodge invertebrates that were collected using the same 110 µm mesh. The colonies were broken with a hammer to liberate the enclosed invertebrates, although fracturing did not liberate boring organisms (e.g., *Lithophaga* spp.). The broken colony pieces were rinsed with freshwater, and additional animals were collected on the same 110 µm mesh. All animals obtained from each colony were placed into a 10-cm petri dish and identified and counted while fresh using a dissecting microscope (40 × magnification). Invertebrates were identified to the lowest possible taxonomic level using a taxonomic guide for the local fauna produced by experts [68]. A Kruskal–Wallis rank sum test [64] was used to test for difference in invertebrate abundance (pooled among taxa) between live and dead corals.

To test the hypothesis that the benthic community on the adjacent substratum differed between live and dead *Pocillopora* colonies, the community structure was quantified from the photoquadrats (1 m^2^ area centered on the tagged colonies) recorded in May 2022, August 2022, January 2023, and April 2023. Photoquadrats were analyzed for coverage by benthic space holders using CoralNet software [69] with manual annotations of 100 points randomly located on each 0.5 × 0.5 m photoquadrat (400 random points per tagged colony). Space holders were resolved to seven functional groups: macroalgae, coral (pooled scleractinian and hydrocoral), crustose coralline algae, non-crustose coralline algae (mostly peyssonnelids), turf, sand, and “other” (i.e., cyanobacteria, anemones, COTs). Benthic community structure was compared between live and dead corals using the mean coverage from the four photoquadrats around each coral as statistical replicates. Multivariate community structure was analyzed using PERMANOVA (PRIMER 7, PERMANOVA+ [70]) in which time and colony type (dead vs live) were fixed effects, time (4 levels) was a repeated measures effect and Type III sum of squares were employed. The data frame was trimmed to retain orthogonal data with 25 live corals and 2 dead corals. Percent cover was log(x + 1) transformed prior to preparing a resemblance matrix using Bray–Curtis dissimilarities. PERMANOVA compares the location of the centroids visualized in two dimensional ordinations and PERMDISP [PERMANOVA+] was used to test for equal dispersions among levels of the main effects, which is an assumption of PERMANOVA.

### Assessment of hollowed out skeleton branches

While completing surveys in 2022, some naturally broken branches of dead *Pocillopora* coralla were internally hollowed along their length (Fig 1D). To quantify the extent to which skeleton branches were hollowed out, a coarse qualitative “crush assay” was developed in which dead branches were laterally compressed with pliers by the same diver for all surveys, *in situ* using SCUBA. The diver applied pressure by hand (~ 5 cm below the tip of the branch) to each branch and the crush assay identified extreme cases of hollowed out skeleton branches while leaving more solid and less diagenetically altered branches unaffected. The extent of hollowing through the whole branch down to the basal region was not assessed due to logistical constraints. Moreover, because most of the dead *Pocillopora* colonies were derived from larger adult colonies with many branches [62,63], it was often impossible to reach more basal parts of the branches to test for hollowing. The crush assay was employed in November 2022, April 2023, and May 2024. To evaluate the number of hollowed out branches on a single colony, 30 dead colonies of *Pocillopora* were randomly selected along a 30-m transect adjacent to a permanently marked phototransect (described above) at LTER 1, and all branches on each selected colony were subjected to the crush assay. The number of hollowed out branches was expressed as a percentage of all branches on each colony and averaged among colonies to quantify the number of branches in each colony that were hollow.

To determine how many colonies were affected by branch hollowing, dead colonies of *Pocillopora* (n = 120) were randomly selected at LTER 2, and a single branch was randomly selected for application of the crush assay. The proportion of colonies on which a randomly selected branch was found to be hollowed out was expressed as a percentage of the colonies sampled. By 2023, the hollow branches that had naturally fractured had become sufficiently common to be detected in the long-term annual photoquadrats (0.5 × 0.5 m, n = 40) used to quantify benthic community structure (e.g., [49]). Exposed hollow branches of *Pocillopora* were counted in each photoquadrat and expressed as a percentage of the total number of combined solid and hollowed out dead branches.

### Sampling, petrographic thin sectioning and high-resolution microscopy

Dead samples of hollowed out *Pocillopora* skeleton branches were collected in April 2023 at 10-m depth from fore reef depositional environments [71] on the north shore of Moorea (S1 Fig). In 2023, six randomly collected and randomly oriented skeleton branches (each ~ 5 cm in length) were collected. Individual hollowed out skeleton branches from small *Pocillopora* colonies (Fig 1D) were removed using a hammer and clean steel chisel with gloved hands. Moist (but not preserved) samples were stored for two days at 4^o^C in Moorea before being shipped to Illinois, where they were soaked for several weeks in dilute 1.5% sodium hypochlorite bleach to remove encrusting organisms without diagenetically altering skeleton mineralogy, structure and composition [72].

Samples were air dried in a clean room, after which each hollowed out branch was cut both latitudinally and longitudinally on a diamond embedded tile saw to prepare billets for thin sectioning. Ultrathin 25 μm-thick sections were prepared at Wagner Petrographic, Linden, Utah. Each thin section was impregnated with clear epoxy, doubly polished and mounted onto standard-sized petrographic glass slides. Unstained petrographic thin sections were analyzed using bright field (BF), circular polarization (CPOL), and super resolution autofluorescence (SRAF) microscopy, as described in previous literature [73–75]. All microscopy analyses were completed on a custom designed Carl Zeiss LSM 980 Spectral NLO Airyscan II Super Resolution system (Carl Zeiss, Oberkochen, Germany) housed in the University of Illinois Urbana-Campaign Roy J. Carver Biotechnology Center.

### Determination of changes in live coral cover over time

To compare the changes in coral cover following the 2010 cyclone and 2019 marine heatwave disturbances, annually sampled photoquadrats at LTER 1 and LTER 2 (described above) were analyzed for five years following both events (2010–2015 and 2019–2024). We report the percentage cover of *Pocillopora*, all other corals pooled among taxa (scleractinians and the hydrocoral *Millepora* sp.), and macroalgae. Percent coral cover and *Pocillopora* cover were pooled and arcsine transformed and univariate ANCOVA [64] was used to compare the rate of change in cover over time between the 5-year periods post two disturbances. The cover of macroalgae was not statistically analyzed as it was not the subject of the present study.

## Results

### Fate of living and dead *Pocillopora* coralla

Out of the 35 colonies tagged in May 2022 to evaluate detachment, 86–97% were found over the year sampled. Of the 30 live colonies tagged in May 2022, 27 were found by the end of the project in April 2023, and all were attached to the reef; the other three were assumed to have broken from the reef (10% detachment). Of the 5 dead colonies tagged in May 2022, 3 were found by the end of the project in April 2023, still attached to the reef, with an assumed loss of the missing 2 (20% detached). Using Fisher’s exact test, there was no significant association of the number of colonies by type (alive vs dead when tagged) and time of survey (df = 3, P = 0.964).

The benthic space occupied around corals was compared between 30 live and 5 dead tagged colonies in May 2022 and subsequently assessed in August 2022, January 2023, and April 2023. Percent cover of vacant space started at 5.1 ± 0.2% in May 2022 and slightly increased by April 2023 to 7.0 ± 0.2%, but there was no significant temporal effect on the percent cover of vacant space (z = 1.493, p = 0.135) surrounding the tagged colonies. Additionally, there was no significant effect of tagged colony condition (i.e., live vs dead, z = 1.248, p = 0.212), and no significant interaction between Condition and Time (p > 0.2).

### Quantification of invertebrates associated with live and dead *Pocillopora* coralla

**Fig 2:**
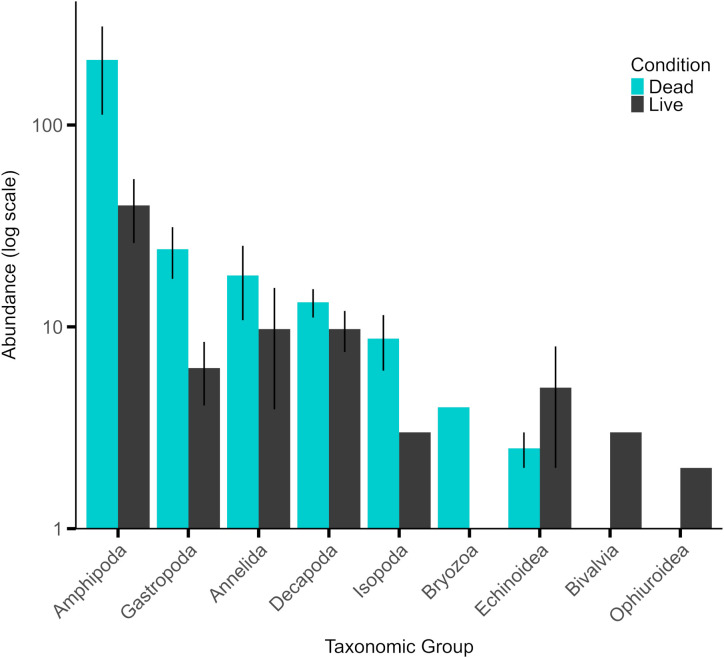
Invertebrates associated with dead and live *Pocillopora* colonies from 10-m depth. Bar graph shows mean raw abundances (± SE, n = 4 per colony condition) of invertebrates collected from *Pocillopora* from the fore reef in 2022–2023. The y-axis is displayed on a logarithmic scale to better visualize differences across taxa with large differences in mean abundances. To ensure visibility of low abundance values (e.g., < 1 animal colony^-1^), a constant (1) was added to all abundance values. Taxonomic groups with only one observation per condition do not have error bars.

A wide variety of invertebrates were found associated with 20-cm diameter live and dead colonies of *Pocillopora*, and they occurred at higher abundances among dead versus live colonies. These invertebrates represented 5 phyla, 6 classes, and 3 orders (Table 1), with dead colonies containing 155 ± 9 animals colony^-1^ and live colonies containing 28 ± 3 animals colony^-1^ (mean ± SE, pooled among taxa); these differences were statistically significant (Fig 2, Kruskal–Wallis χ² = 4.467, df = 1, p = 0.035). The abundance of amphipods was 7-fold greater in dead (209 ± 6 animals colony^-1^) versus live (39 ± 2 animals colony^-1^) (mean ± SE, n = 4 per condition) colonies.

**Table 1:** Taxonomic rank of associated invertebrates with live and dead *Pocillopora.*

Phylum	Class	Order
Arthropoda	Crustacea	Amphipoda
	Malacostraca	Decapoda
		Isopoda
Annelida		
Bryozoa		
Echinodermata	Echinoidea	
	Ophiuroidea	
Mollusca	Bivalvia	
	Gastropoda	

Invertebrates collected from live and dead coral colonies were identified to the lowest possible taxonomic resolution but grouped for analysis into Phylum, Class, or Order.

The benthic communities adjacent to dead and live colonies of *Pocillopora* were spatially dominated by algal turf (38.3 ± 0.8% cover) and macroalgae (mostly *Lobophora variegata* [32.9 ± 0.7%] and *Asparagopsis taxiformis* [14.5 ± 0.7%]) in all sampling months (S2 Fig). Multivariate benthic community structure differed between live and dead corals (Pseudo-F = 2.790, df = 1,399, p_perm_ = 0.033), and over time (Pseudo-F = 13.869, df = 3,399, p_perm_ = 0.001) but there was no interaction between the coral status and time (Pseudo-F = 44.842, df = 3,399, p_perm_ = 0.208). Dispersion differed between live and dead corals (F = 15.341, df = 1,430, p_perm_ = 0.003), but not among times (F = 2.455, df = 3,428, p_perm_ = 0.110), suggesting that the significant effect of coral status should be interpreted with caution. The difference in mean dispersion between live (18.35 ± 0.34, n = 400) and dead (13.55 ± 0.88, n = 32) corals likely reflected the unbalanced experimental design.

### Evaluation of remnant hollowed out *Pocillopora* skeleton branches

As described above, the crush assay identified extensively hollowed out skeleton branches within dead colonies of *Pocillopora* (Fig 1D). In November 2022, dead colonies each contained 13–66 branches, of which 5 ± 1% (mean ± SE, n = 30) was hollowed out. In 2023, application of the crush assay to one randomly selected branch on 120 colonies revealed 49 hollowed out branches (41%). Concurrently, photoquadrats (0.5 × 0.5 m) contained 94 ± 5 dead *Pocillopora* branches (mean ± SE, n = 77), of which 45.5 ± 0.4% was hollowed out. In May 2024, many of the dead colonies were found with broken branches, representing damage caused by a severe storm that hit Moorea in February 2024 (K. Scafidi, personal observation). The crush assay was repeated by testing all 15–79 branches of individual dead coralla, with 5 ± 1% (mean ± SE, n = 26) hollowed out.

**Fig 3:**
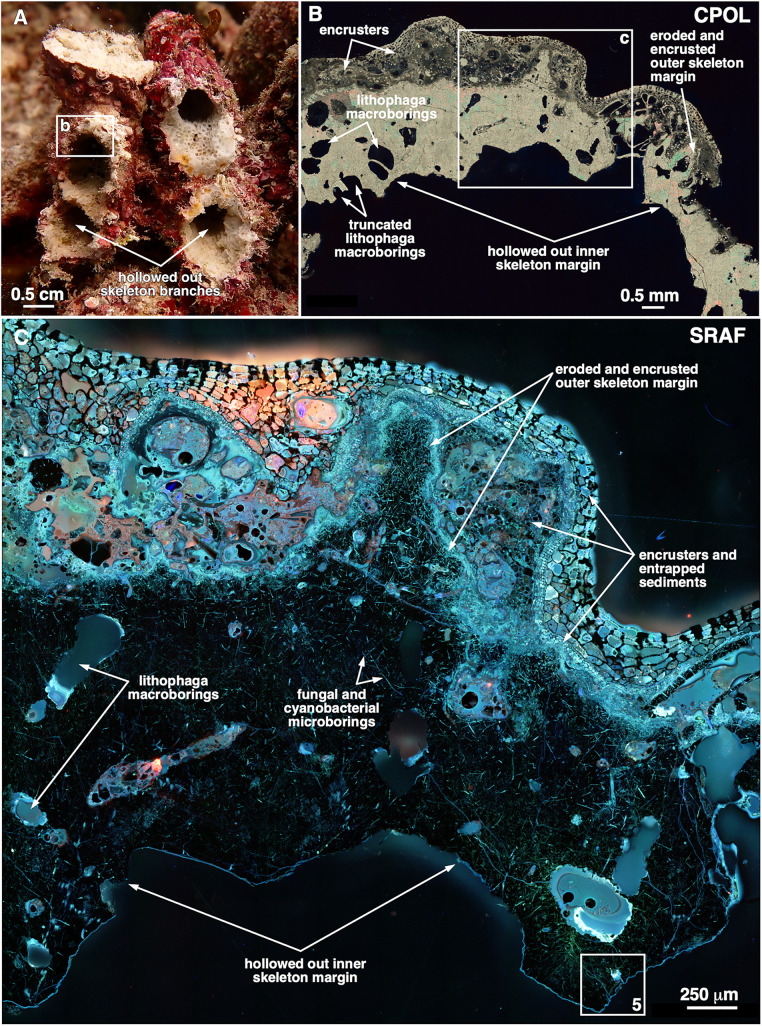
Structure and composition of remnant hollowed out *Pocillopora* skeleton branches. **(A)** Field photograph of hollowed out eroded and encrusted skeleton branches at 10-m water depth. **(B)** Circular polarization (CPOL) microscopy image of the area of heavily eroded and encrusted remnant skeleton shown in white box in **A. (C)** Super resolution autofluorescence (SRAF) microscopy enlargement image of the area of heavily eroded and encrusted remnant skeleton shown in white box in **(B)**. Note that the original coral skeleton is dark and exhibits no SRAF emissions other than from organic matter preserved within fungal hyphae borings and sediments adjacent to the skeleton margin.

**Fig 4:**
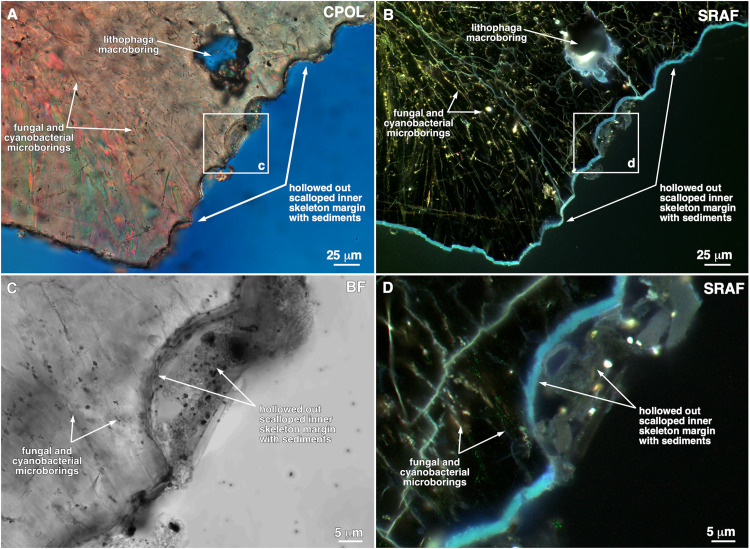
Structure and composition of scalloped inner margin of remnant hollowed out *Pocillopora* skeleton branches. Microscopy images are enlargements of remnant skeleton area shown by white box in Fig 3C. **(A and B)** Paired circular polarization (CPOL) and super resolution autofluorescence (SRAF) images of the inner scalloped margin of the remnant hollowed out skeleton. **(C and D)** Paired bright field (CPOL) and super resolution autofluorescence (SRAF) microscopy images of the areas of scalloped inner margin of the remnant skeleton shown by white boxes in **A** and **B.** Note that the original coral skeleton is dark and exhibits no SRAF emissions in **B** and **D** other than from organic matter preserved within fungal hyphae borings and sediments adjacent to the skeleton margin.

Observations from the reefs of Moorea (described above) (Figs 1 and 3A), combined with high resolution microscopy of dead branch samples (Fig 3A,B), indicate that the outermost surface of each hollowed out *Pocillopora* skeleton branch consisted of an irregular and heavily eroded surface encrusted by coralline algae, serpulid tube worms, sponges, and bryozoans that trapped and bound carbonate sediments (Fig 3A,B). In addition, the remnant coral skeletons exhibited extensive fungal and cyanobacterial microborings and *Lithophaga* and possibly sponge macroborings (Figs 3A,B and 4), which in combination with grazing activity and storms resulted in high rates of calcium carbonate erosion [76–80]. The innermost margin of each hollowed out *Pocillopora* branch exhibited angular to arcuate surfaces (Fig 3B,C). At higher magnification, these inner margins ranged from having relatively smooth, nearly linear edges, to distinct and clean-cut scalloped patterns, with each scallop on the scale of 20–30 μm in diameter with almost beveled edges (Figs 4A–D). Some *Lithophaga* macroborings were truncated by the inner margin of the remnant skeletons (Fig 3B), indicating that the macroborings formed prior to the hollowing out process.

### Comparisons of coral reef community structure

**Fig 5:**
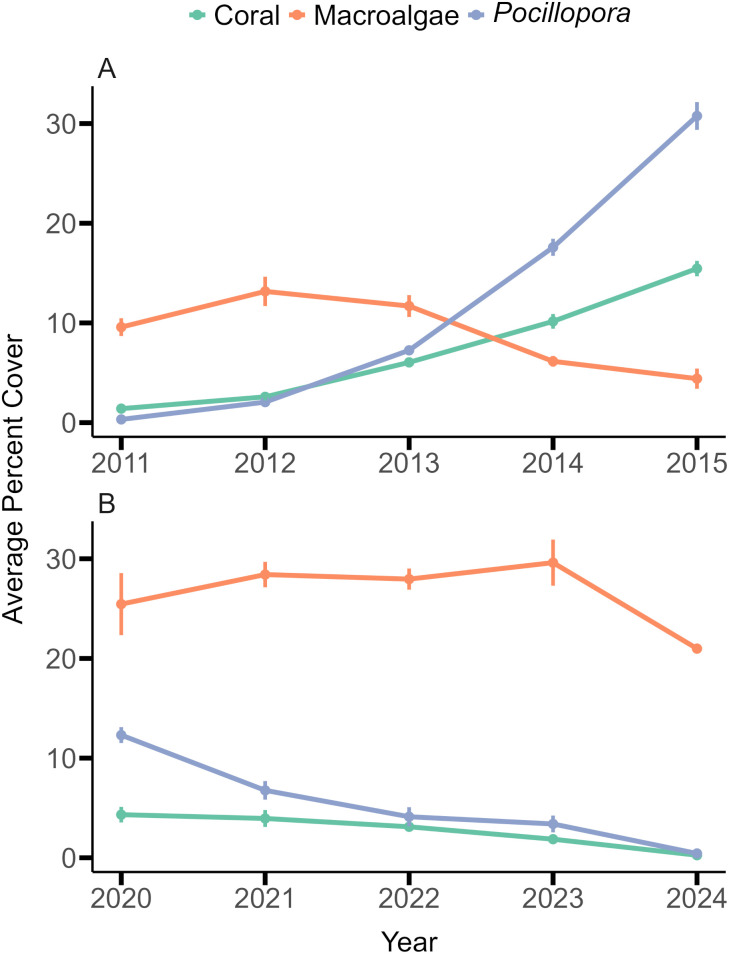
Comparison of changes in community structure 5 years after two major disturbance events. Benthic community structure at 10-m depth on the north shore fore reef of Moorea following corallivory by *Acanthaster* spp. and cyclone Oli in 2010 **(A)** and bleaching in 2019 **(B)**. Both graphs show mean (± SE, n = 77–80 year^-1^) percentage cover over a 5-year period for all corals and *Millepora* sp. (pooled among taxa), *Pocillopora*, and macroalgae.

Following the two sets of disturbances that culminated in low coral cover in 2010 and 2019, the coral reef communities changed in different ways over the subsequent five years (Fig 5). The cover of coral (pooled among taxa, including *Millepora* sp.) and *Pocillopora* increased from 2011–2015, but decreased from 2020–2024. ANCOVA showed that the cover of coral and *Pocillopora* changed over time at different rates for the two five-year periods following the disturbances (F = 126.755, df = 1,6, P < 0.001).

## Discussion

### Overview

With coral cover declining on tropical reefs throughout the world [81], understanding the factors killing corals is profoundly important, as is evaluating the conditions under which coral recovery might occur [82]. These issues have been at the forefront of coral reef science for decades [40,83,84], but attention recently has turned to the legacy effects of dead coral skeletons [36,38,85]. In most cases these are created by rubble from dead corals [36,38], but they can also be produced by dead-in-place coral colonies, as occurs following bleaching, outbreaks of predatory sea stars, and coral diseases [86–88]. These events favor rubble formation when they are followed by storms (e.g., in Moorea [44,51]). The present study focused on the consequences of vast numbers of dead-in-place branching corals that were created on the fore reef of Moorea by bleaching in 2019 [62,63] that was not followed by a major storm.

Following bleaching in 2019, dead *Pocillopora* persisted in growth position on the fore reef for five years where they became encrusted by algal turf, *L. variegata,* and other macroalgae. They accumulated invertebrates among their branches, although these animals were not associated with changes in the benthic community structure adjacent to the colonies. Unlike the aftermath of the previous most recent large-scale disturbance affecting the reefs of Moorea [44,48,51], over 2019–2025 the decimated coral community has not recovered in part because the dead *Pocillopora* deterred coral recruitment. Within three years of death, hollowing of *Pocillopora* branches was detected, with this outcome potentially mediated by the scaffolding of encrusting taxa on the outer surface of dead branches. Hollowing weakened the branches and increased the ease with which they could be broken, and this contributed to rubble formation following the effects of tropical storm Nat in February 2024. The persistence of dead *Pocillopora* colonies created a five-year hiatus in the commencement of coral community recovery (see also [87,88]), underscoring the importance of the legacy effects of dead coral colonies [85] as well as the role of branch hollowing in bringing these effects to a close.

To evaluate the legacy effects of dead *Pocillopora* on the fore reef of Moorea, we quantified the rate at which dead colonies were removed from the reef. By 2022, dead colonies were heavily encrusted (Fig 1) with taxa that filled much of the space between dead branches. The occlusion of void space reduced the ability of seawater to flow among the branches [89] and increased the potential for flow to skim across the outer surface of the colonies [89,90]. Large areas of dead-in-place branching corals have been reported following bleaching around Lizard Island, Great Barrier Reef [36], other parts of the Great Barrier Reef [91], and many other locations. Similar to the observation reported here, the dead-in-place colonies typically became encrusted by algal turfs and algae belonging to several functional groups including macroalgae [91]; *Lobophora* spp. is a common component of the macroalgal group [92]. However, few studies have evaluated the fate of dead-in-place coral colonies [91,93–95] particularly for branching species [36,96]. In Moorea, colonies of *Pocillopora* that were killed by COTs in the backreef from 2003–2010 [61] eroded and broke from the substratum within 3–5 years, killing corals that recruited to their surfaces [96]. One study evaluated the fate of 143 branching corals on the shallow (≤ 4-m depth) reefs of Lizard Island, Great Barrier Reef, following bleaching in 2016, 2017 and 2020, and they found that 80% of the dead corals eroded and disappeared within 60 months [36]. The “half-life” of dead branching corals was 40 months. Where interaction between branching corals and ambient flow have been studied (e.g., [97]), the likelihood of breakage is determined in part by the extent to which branches serve as roughness elements. Therefore, seawater flow that skims the surface of branching coral colonies with occluded interstices among their branches is less likely to cause colony breakage.

In Moorea, dead colonies of *Pocillopora* persisted on the reef during the study period, and the results indicate some variation in vacant space over time within the 1 m^2^ plots, but the condition of the tagged colonies does not contribute to the amount of vacant space surrounding the colonies. These results are consistent with observations (by K. Scafidi) from larger areas on the fore reefs of Moorea in 2022 and 2023, and they support the inference that dead-in-place *Pocillopora* persist for years in the absence of storms. Aspects of this assertion have been supported from analyses of coral colony erosion on the Great Barrier Reef [36], although only 20% of dead branching colonies on the shallow reefs of Lizard Island persisted > 5 years. The persistence of dead-in-place branching corals supports the possibility that dead branching corals intensify the hysteresis associated with the alternative stable state of macroalgal dominance [87,88]. Analysis of the fore reef of Moorea after the bleaching event of 2019 suggested the intensification of hysteresis was facilitated by refuges among dead coral branches in which macroalgae could avoid fish herbivores [87]. Evidence for the intensification of hysteresis in the coral-macroalgal transition remains theoretical [87] or anecdotal (present study, [88]), although when dead *Pocillopora* were removed from the fore reef in 2019, coral recruitment increased [88], suggesting a phase reversal could be initiated through removal of dead colonies.

However, coral recruitment on the fore reef of Moorea also increased in 2014 when live *Pocillopora* were removed, supporting an inference of density dependent *Pocillopora* recruitment [51]. The mechanism favoring enhanced coral recruitment when dead colonies of *Pocillopora* are removed (i.e., as in [88]) is unlikely to solely be the macroalgae that colonize dead coral colonies. As *Lobophora* spp. can deter coral recruitment (e.g., in Palau, [98]), and this alga was abundant on dead *Pocillopora* in the present study, it might be valuable to conduct experiments to distinguish the effects of macroalgal inhibition (sensu [88,98]) from space limitation caused by congeneric colonies of adult corals (sensu [51]) as mechanisms preventing pocilloporid recruitment. As pocilloporid recruits were common on settlement tiles on the fore reef of Moorea in the first few years following bleaching (PJ Edmunds [Unpublished]), low coral recruitment among dead *Pocillopora* as detected in the present study is unlikely to reflect a lack of coral propagules.

To gain insight into the implications of dead *Pocillopora* on the fore reef of Moorea, we quantified invertebrates among their branches. Live *Pocillopora* contain diverse assemblages of motile invertebrates, with *Trapezia* crabs and alpheid shrimps among the best known [99–101], although many other invertebrates have been described in *Pocillopora* colonies on the Great Barrier Reef [100], and Moorea [102]. In the present study, invertebrates associated with dead *Pocillopora* were 7-fold more abundant, and 59% taxonomically richer, than those associated with live congenerics. These analyses were restricted to a common size of *Pocillopora* colonies (i.e., ~ 20-cm diameter), leaving open the possibility that the discrepancy in invertebrates associated with dead versus live colonies might vary with colony size [103]. Regardless of this possibility, the high abundance of amphipods within dead *Pocillopora* may have ecological implications because amphipods are voracious herbivores [53] and will forage on less palatable food if their preferential kind is unavailable [104]. These trends led us to hypothesize that the benthic communities adjacent to dead and live *Pocillopora* would differ through the foraging of invertebrates living among these colonies, however, statistical analysis did not support this hypothesis.

While assessing the fate of dead and live colonies of *Pocillopora*, hollow branches (Fig 1D) were unexpected based on observations of dead branching corals eroding largely from the outer surfaces of the colony [36]. Such effects might act through chemical dissolution [105] or bioerosion from invertebrate and fish corallivory [106]. Research with corals on the northern Great Barrier Reef that were killed by bleaching in 2016, suggested that the formation of a microbial layer on the outer surface of dead corals drives branch dissolution [107], although the role of seawater solution carbonate geochemistry was not included in the analysis and there was no report of hollowed out coral branches (cf. Figs 1 and 3). To our knowledge, there are no previous records of coral branches being hollowed out on the seafloor following death, which suggests the bleaching of 2019, and the context of coral death of the following years, was unusual in Moorea. Although we have not conducted exhaustive surveys throughout French Polynesia, anecdotal observations on Huahine and Manihi in 2023 (by K. Scafidi) also revealed hollowed out branches on *Pocillopora* colonies in the fore reef habitat.

Our analysis of *Pocillopora* branches by a “crush assay” revealed hollow branches and estimates of their prevalence over time. In November 2022, 5% of the branches on each colony were hollow, and five months later in 2023, 40% of colonies tested positive for a hollow branch when one branch was sampled. This suggests that hollow branches became more common from 2022 to 2023 and had become sufficiently abundant to be detected (as fractured “tubes”) in 2024 in the photoquadrats used to quantify community structure. While the detection of only 5% of colonies with hollow branches in May 2024 is inconsistent with the hypothesis of progressive hollowing after 2022, we suspect that many of the hollow branches were removed by tropical storm Nat that hit Moorea in February 2024. The findings of the crush assay are conservative estimates of the most extreme hollowing of the branch portions that were compressed.

No previously published work has reported the type of hollowed out coral skeleton branches analyzed in the present study. However, a hollowed out coralla of a massive coral (*Porites*) was described from Heron Island, Great Barrier Reef and was hypothesized to have formed by sponge endolithic borings [108], although detailed analyses were not completed. As a result, why *Pocillopora* skeleton branches became hollow on the reefs of Moorea, and why similar effects have not previously been reported on reefs around the world, remains unclear. *Pocillopora* coralla exhibit skeletal morphological plasticity and life history characteristics that associate with changes in water depth, the quantity of light, and seawater flow [109–113]. The aragonitic (CaCO_3_) skeletal ultrastructure of *Pocillopora* [114], its origin as nascent high-Mg calcite (CaCO_3_) that transitions into aragonite [115,116], diurnal patterns of skeletal formation [117], and associated dissolution kinetics [118] have been extensively studied. The tightly intergrown bundled trabeculae and septa [114] of *Pocillopora* forms the highest bulk skeletal density of any Pacific coral, reaching 2.1 ± 0.3 g cm^-3^ [119], with wall extension rates of 4−20 μm d^-1^ [120]. Together, these attributes make *Pocillopora* one of the most resistant of the Pacific coral skeletons to dissolution and ocean acidification [118], suggesting that hollowed out skeleton branches should be unlikely to occur.

However, the high-resolution microscopy examination conducted here of the remnant innermost walls of the hollowed out skeleton branches (Figs 3–4) shed preliminary light on their mode of formation. On the millimeter to centimeter-scale (Fig 3), the hollowed out inner remnant skeleton margin exhibits broad arcuate-to-linear-to-angular shaped edges that are not consistent with the type of marine carbonate grain margin geometries commonly associated with typical seafloor dissolution and diagenesis [121,122]. Evidence against a pure dissolution process is further provided by the 10’s μm-scale scalloped margins with nearly beveled edges (Figs 4A–D) that were common within the millimeter-to-centimeter scale arcuate shapes on the inner margin of the hollowed void (Fig 3B,C). The scale and geometry of these inner remnant skeleton edges are most consistent with the brittle fracture of the dense aragonite skeleton by bioeroders and grazers, however experimental confirmation was beyond the scope of the present study. Furthermore, as has been commonly observed in marine carbonate hardground diagenesis [121,122], the variably thick continuous layers of marine encrusters on the outermost surface of the branches, as well as the sediments they bind and potential marine cementation, serve to structurally reinforce and maintain the branched shape of the hollowed out skeleton branches and prevent their collapse (Figs 3A–C). In early 2025 we conducted a preliminary experiment in which we tested for *in situ* (12-m depth) hollowing of freshly killed *Pocillopora* branches that either were naturally allowed to accumulate encrusting taxa or were periodically brushed to remove encrusting taxa. Over six months, one of four naturally encrusted branches started to hollow, whereas none of four brushed branches began to hollow. Although preliminary, the outcome provides some support for our assertion that the sheeting of dead *Pocillopora* branches by encrusting taxa is functionally related to the onset of internal hollowing.

Following cyclone Oli in 2010 and bleaching in 2019, the trajectories of changing coral communities on the fore reef of Moorea differed over 2011–2015 versus 2020–2024 (Fig 5). Over 2011–2015, there was rapid and extensive recovery (at 11% y^-1^) of the coral community, but over 2020–2024, coral cover continued to decline (at 5% y^-1^). Evaluating the factors causing trajectories of coral cover to vary has become an important objective of coral reef science, with emphasis placed on the lack of recovery that characterizes the current state of many coral reefs [81]. Crustose coralline algae (CCA) for example, contribute to coral reef recovery following disturbances by binding coral rubble through cementation and promoting coral larvae settlement [123]. Immediately following the 2010 cyclone in Moorea, CCA cover was recorded at 28% [124] and during the subsequent coral recovery CCA cover decreased to < 10% [125]. However, after bleaching in 2019, CCA cover remained < 11% while algal turf and *L. variegata* combined covered 67–77% of the reef. There are numerous conditions that lead to coral death and suppress subsequent recovery (e.g., [126]), and these are frequently interpreted in the context of alternative stable states and hysteresis that increases the difficulty of phase reversal back to coral dominance [127]. Since 2019, the fore reef of Moorea has made a compelling case for the importance of the legacy effects of dead corals in deferring coral community recovery by intensifying the hysteresis of alternative stable state formation [87,88]. Kopecky et al. [87] demonstrated that these legacy effects can be mediated through refuges among coral branches that allow macroalgae to escape herbivory, and here we reveal the consequences of encrusting organisms in promoting internal hollowing and temporal synchrony of colony loss through breakage.

### Summary

Long-term analyses of coral reefs are required to provide ecological meaning to the coral reef crisis by placing it in the context of the responses to previous disturbances. Even with decades of data describing coral reef community structure at multiple locations (e.g., [128,129]), ecological surprises (sensu [130]) continue to be revealed. The ability of science to accurately predict the outcomes of ecological events on coral reefs remains incomplete. Such was the case with the reefs of Moorea following bleaching in 2019 [62], where the expectation of rapid coral community recovery following a major disturbance [44] did not match reality. As it has become clear from decades of studies of disturbances on coral reefs [131,132] and other ecosystems [133,134], the recent history of the communities plays a large role in determining their response to new disturbances [134,135]. These effects have been described as “ecological memory” [136,137], with examples provided by the effects of marine heatwaves on coral reefs that have impacts depending on the previous marine heatwaves [138–140], and legacy effects and subtle examples by thermal priming that reduces the subsequent impacts of high temperature [141].

In Moorea, the confluence of several events favored the outcomes observed on the fore reef from 2020 to 2024. First, rapid coral community recovery from 2010–2019 with high abundance of *Pocillopora* created a community with 58% coral cover that was susceptible to bleaching [62]. Second, seawater warming and bleaching in 2019 created dead *Pocillopora* colonies that remained in growth position in the absence of severe storms. Third, macroalgae rapidly populated the dead *Pocillopora*, within the voids among branches protecting macroalgae from herbivory (cf. [88]) and intensifying hysteresis favoring algal dominance. Fourth, growths of encrusting organisms on dead branches allowed internal hollowing to occur, delaying the breakage and clearing of rubble to initiate coral community recovery. The inability to accurately predict this chronology rendered the outcome of the 2019 bleaching an ecological surprise, the causes of which are only beginning to be understood (present study, [87,88]).

## Supporting information

S1 Fig Map of Moorea, French Polynesia. A map of the island of Moorea, French Polynesia and the focal area of the north shore (A), showing the study sites (LTER 1 & LTER 2) on the fore reef (B). Island outline is from GADM (v4.1) and reef crest geomorphic data is from Allen Coral Atlas [54,55]; spatial layers were processed and visualized in R [64].
(TIF)

S2 Fig Heatmap describing the benthic community structure changes grouped by year in surrounding tagged *Pocillopora.* Analyzed areas (1 m^2^) surrounding dead (n = 5) and live (n = 30) *Pocillopora* colonies with functional groups shown represent at least 5% of cover. All coral, including the hydrocoral *Millepora* sp., were pooled into a functional group “Coral” that covered < 1% of the benthos.
(TIF)
