# Supplementary figures and images for: Remnant hollowed out dead coral skeleton branches defer coral community recovery
Source: PLoS One. 2026 Mar 11;21(3):e0339527. doi: 10.1371/journal.pone.0339527 (PMC12978499; doi:10.1371/journal.pone.0339527)

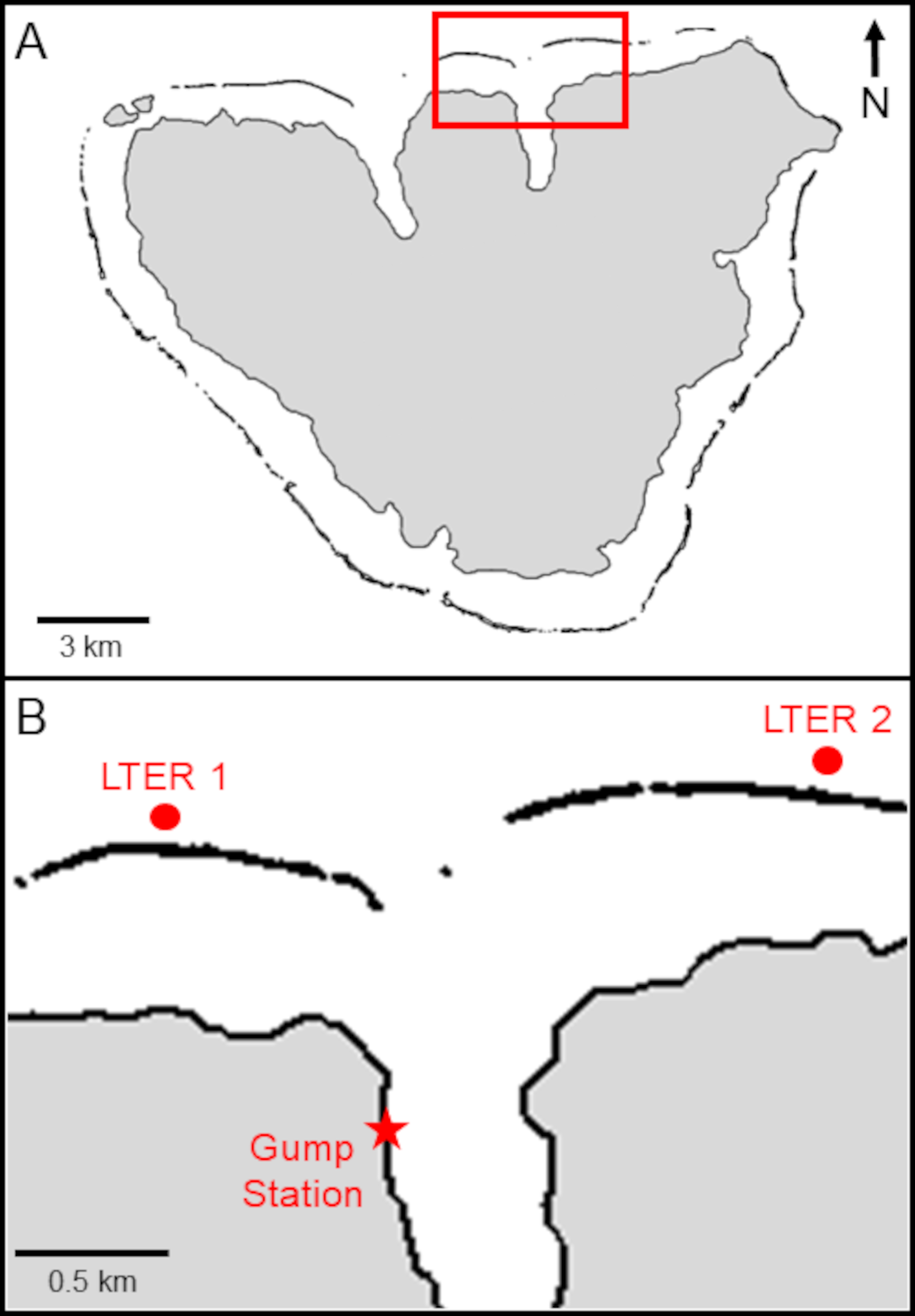

Supplement: S1 Fig — A map of the island of Moorea, French Polynesia and the focal area of the north shore (A), showing the study sites (LTER 1 & LTER 2) on the fore reef (B). Island outline is from GADM (v4.1) and reef crest geomorphic data is from Allen Coral Atlas [54,55]; spatial layers were processed and visualized in R [64]. (TIF) [file pone.0339527.s001.tif]

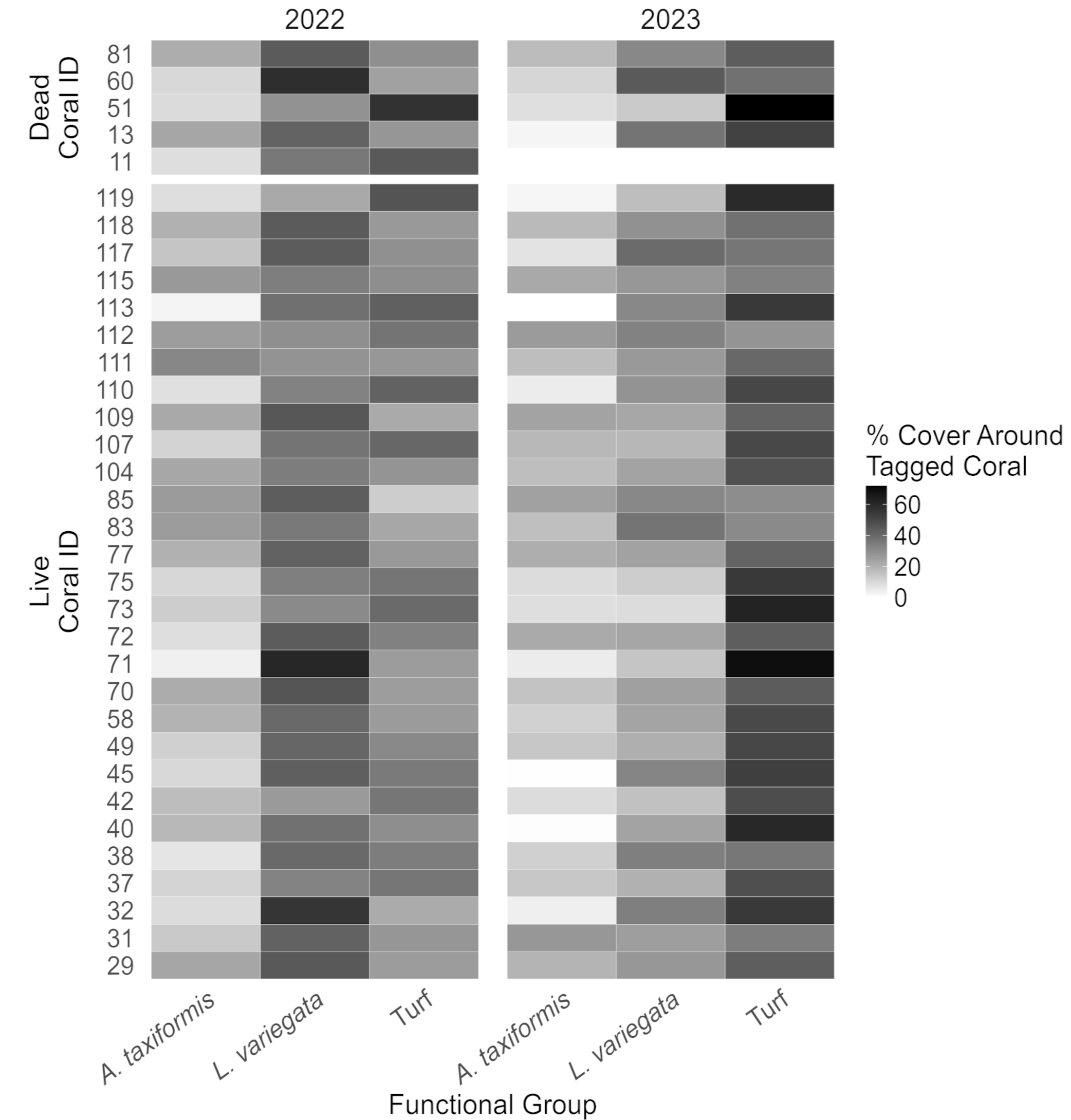

Supplement: S2 Fig — Analyzed areas (1 m2) surrounding dead (n = 5) and live (n = 30) Pocillopora colonies with functional groups shown represent at least 5% of cover. All coral, including the hydrocoral Millepora sp., were pooled into a functional group “Coral” that covered < 1% of the benthos. (TIF) [file pone.0339527.s002.tif]
